# The Orange Carotenoid Protein Triggers Cyanobacterial Photoprotection by Quenching Bilins via a Structural Switch of Its Carotenoid

**DOI:** 10.1021/jacs.4c06695

**Published:** 2024-07-26

**Authors:** Nicoletta Liguori, Ivo H.M. van Stokkum, Fernando Muzzopappa, John T. M. Kennis, Diana Kirilovsky, Roberta Croce

**Affiliations:** † Department of Physics and Astronomy and Institute for Lasers, Life and Biophotonics, Faculty of Science, Vrije Universiteit Amsterdam, de Boelelaan 1081, 1081 HV Amsterdam, The Netherlands; ‡ Institute for Integrative Biology of the Cell (I2BC), CNRS, CEA, Université Paris-Sud, Université Paris-Saclay, 91198 Gif sur Yvette ,France

## Abstract

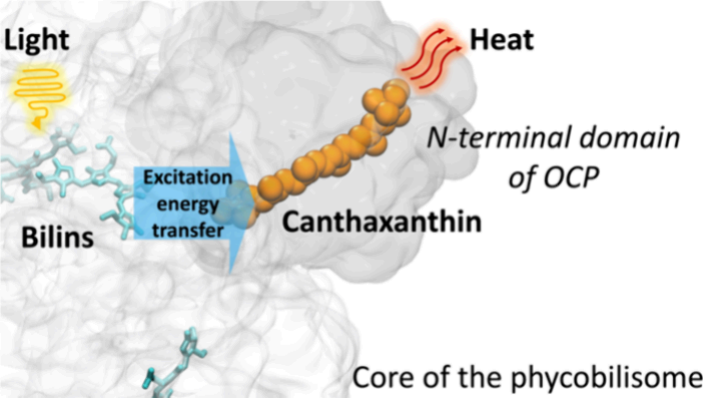

Cyanobacteria were the first microorganisms that released oxygen into the atmosphere billions of years ago. To do it safely under intense sunlight, they developed strategies that prevent photooxidation in the photosynthetic membrane, by regulating the light-harvesting activity of their antenna complexes–the phycobilisomes–via the orange-carotenoid protein (OCP). This water-soluble protein interacts with the phycobilisomes and triggers nonphotochemical quenching (NPQ), a mechanism that safely dissipates overexcitation in the membrane. To date, the mechanism of action of OCP in performing NPQ is unknown. In this work, we performed ultrafast spectroscopy on a minimal NPQ system composed of the active domain of OCP bound to the phycobilisome core. The use of this system allowed us to disentangle the signal of the carotenoid from that of the bilins. Our results demonstrate that the binding to the phycobilisomes modifies the structure of the ketocarotenoid associated with OCP. We show that this molecular switch activates NPQ, by enabling excitation-energy transfer from the antenna pigments to the ketocarotenoid.

## Introduction

Life under fluctuating light conditions is not without danger for photosynthetic organisms. Sudden spikes in sunlight intensity can saturate the photosynthetic electron transport chain, creating an excess of photoexcitations in the thylakoid membrane, which may ultimately lead to photooxidative damage. Throughout evolution, however, photosynthetic organisms provided themselves with an effective set of molecular tools, which allows them to dissipate the excess excitation energy as heat. One of these photoprotective processes is known as nonphotochemical quenching (NPQ) and, in distinct forms, is present in plants, algae and cyanobacteria.^1,2^

**Figure 1:**
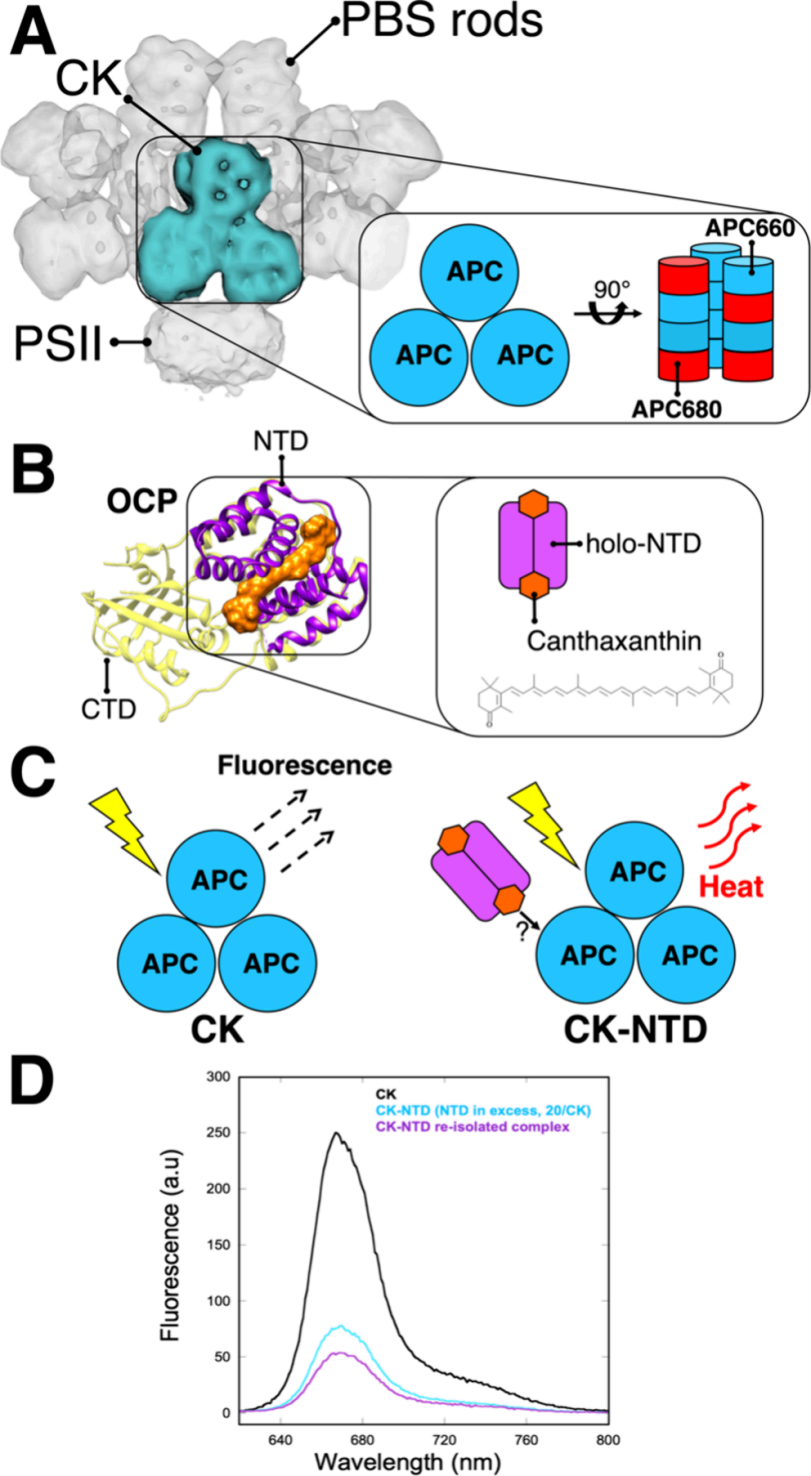
The core of the phycobilisomes and the orange carotenoid protein (OCP). (A) In the black quadrant on the right, a model of the isolated cyanobacterial allophycocyanin (APC) core cylinders (CK) is reported. Each cylinder contains 4 disks, for a total of 72 APC pigments. The basal disks contain a total of 6 red APC absorbing at 680 nm (APC680), while the rest absorbs at 660 nm (APC660). On the left, the structure of a complete phycobilisome, including the peripheral rods, in complex with photosystem II (PSII) resolved from *Anabaena* sp. strain PCC 7120^57^ is depicted (Electron Microscopy Data Bank, 2822). (B) In the black quadrant on the right, a model of the N-terminal domain (NTD) of OCP in its active state is reported. The NTD protein domain, modeled in purple, binds a carotenoid (holo-NTD). The structure of the NTD carotenoid studied in this work, canthaxanthin, is reported in the quadrant. The structure of inactive OCP is shown in yellow (PDB 5UI2)^10^ on the left and includes the C-terminal domain (CTD), together with the active domain NTD in purple (PDB 4XB4).^15^ (C) On the left, a cartoon of isolated CK set in a light-harvesting state is shown. On the right, the CK-NTD complex, resulting from the association of holo-NTD to CK, is shown (OCP is depicted larger than in real scale for clarity). CK-NTD is strongly quenched. The structure solved in ref (24) shows that the active OCP binds to the core of the PBS (here called CK). The question mark in the CK-NTD cartoon represents the unknown origin of quenching and the unknown effect on the structural dynamics and energetics of canthaxanthin, upon binding of NTD to CK. (D) Steady-state emission spectra of CK (black), CK-NTD (cyan) and after complex reisolation (purple). CK-NTD complex was prepared by mixing 0.05 μM cc CK and NTD 1 μM (20 per CK) in 1.2 M phosphate buffer (final volume was 5 mL) at RT for 10 min. Fluorescence emission spectra of the samples were measured before and after the addition of NTD (the sample was diluted until A655 = 0.036) using an excitation centered at 600 nm. To reisolate the complex, CK-NTD was precipitated by ultracentrifugation at 48,000 rpm at 23 °C for 3 h, two times (the excess of NTD remained in the supernatant).

Cyanobacteria, in particular, are the most abundant oxygenic photoautotrophs on Earth^3^ and, likely, the most ancient organisms that required molecular strategies to prevent photooxidation.^4^ Their light-harvesting machinery consists of >10^3^ kDa supramolecular complexes called phycobilisomes (PBS), composed of different types of phycobiliproteins that covalently bind phycobilin pigments.^5^ A typical structure of a cyanobacterial PBS is shown in Figure 1A. In the case of *Synechocystis* sp. PCC 6803, a model organism for photosynthesis studies in cyanobacteria, PBS are composed of a central core (CK), consisting of 3 cylinders of allophycocyanin (APC), from which several rods of phycocyanin radiate.^6^ PBS are water-soluble, and the assembly of the whole structure (CK plus rods) is arranged by linker proteins. PBS are attached via the two basal APC cylinders to the stromal side of the thylakoid membrane, in functional proximity to the photosystems.^7^ Each APC cylinder consists of 4 disks of trimeric proteins, binding a total of 24 phycocyanobilins. Most phycocyanobilins emit at 660 nm (APC660), while up to 2 pigments in the basal cylinders emit at ≈680 nm (APC680). The latter ones represent the lowest energy sites of the whole PBS and are responsible for funneling the excitation energy to the photosystems (Figure 1A).

The NPQ mechanism in cyanobacteria is activated by strong blue-green light, which triggers the interaction between a carotenoid-binding protein, called orange-carotenoid protein (OCP), and the PBS.^8,9^ OCP is a 35 kDa water-soluble protein composed of an α/β-fold C-terminal domain (CTD) and an α-helical N-terminal domain (NTD), connected via a flexible linker^10^ (Figure 1B). A single ketocarotenoid (typically 3′hydroxyechinenone, canthaxanthin or echinenone)^10−13^ is encapsulated and is stabilized via hydrogen bonds in the C-terminal domain.^10^ OCP is a photoactive protein:^14^ i.e., strong blue-green light is absorbed by the ketocarotenoid, which in turn detaches from CTD and translocates 1.2 nm into the NTD domain.^14−16^ This event is followed by dissociation and separation of NTD and CTD.^17−20^ This final arrangement represents the active form of OCP, which is called OCP^R^, while the inactive form is called OCP^O^. The superscripts R and O refer to the red and orange colors of the two OCP forms. OCP^R^ has been proposed to bind to CK via the carotenoid-binding NTD (holo-NTD),^21^ triggering quenching in the PBS (Figure 1C).^22,23^ This proposal was recently validated by the first cryo-EM structure of a quenched OCP-PBS complex.^24^ This structure shows 4 OCP binding in the form of 2 dimers to the basal and top cylinders of CK.

Although the key players of NPQ in cyanobacteria have been identified,^8,24,25^ key questions remain unanswered to date: i.e., (i) what is the quenching mechanism induced by OCP? (ii) Is a conformational switch of the carotenoid bound to OCP the “ignition key” of NPQ in cyanobacteria? Here we address both points using ultrafast spectroscopy and compartmental model fitting, applied to an in vitro system composed of the 2 domains central to NPQ activation–the active domain of OCP (NTD) and the site of quenching (CK). In the first part of this work, we provide evidence that this system maintains the capacity to activate nonphotochemical quenching without the need of photoactivation and with a minimal number of pigments (76 bilins and 1 carotenoid), allowing the study of photoprotective quenching while minimizing the risk of incurring in power-dependent kinetics due to the excitation of a large, connected antenna system. A full description of the model is presented in the SI. The compartmental model allowed us to extract information on the spectrum and associated lifetime of the quencher and, by determining them, to identify the molecular species responsible for quenching. We conclude by discussing the molecular mechanism that could be responsible for activating quenching in CK-NTD and, more in general, in OCP-dependent nonphotochemical quenching.

## Results and Discussion

### Binding of NTD to CK Generates Strong Quenching

NTD binding canthaxanthin was synthesized in *E. coli*,^12^ while CK was isolated from a mutant of *Synechocystis* PCC 6803 lacking the rods.^26,27^ A complex composed of NTD bound to CK (CK-NTD) was obtained as described in the Methods. As shown by the strong reduction of the fluorescence emission in CK-NTD as compared to CK (Figure 1D), NTD has the capacity to induce quenching in CK. This is indeed expected since NTD was reported to maintain the structural and spectroscopic features of the N-terminal domain of OCP^R15^.

To unravel the origin of the quenching in CK-NTD, ultrafast transient absorption (TA) experiments were conducted on both CK and CK-NTD, and the results were compared. We excited preferentially either APC (630 and 694 nm) or canthaxanthin (520 nm) to determine: (i) which species dissipates the excitation energy of APC and (ii) what is the effect of the binding of NTD to CK on the energetics of canthaxanthin.

**Figure 2:**
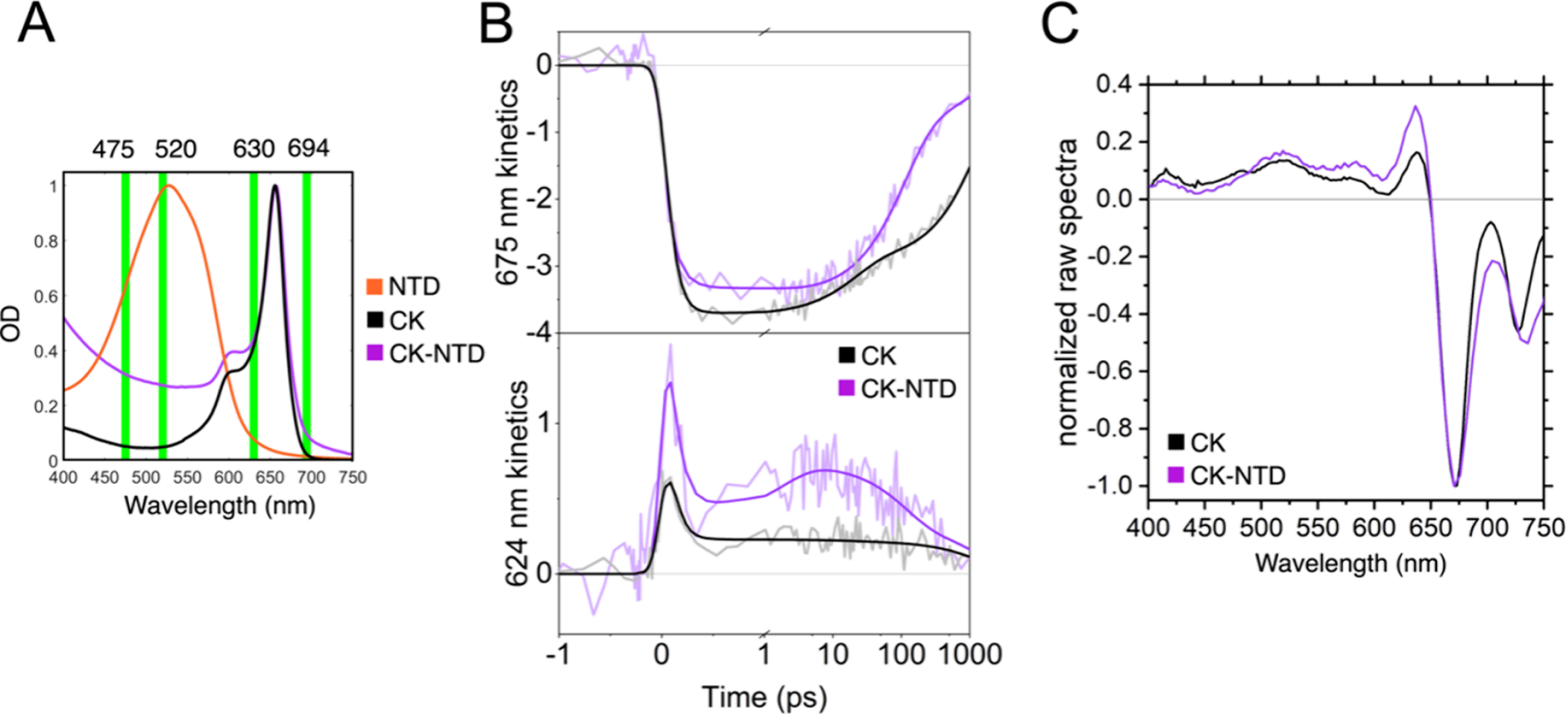
Binding of NTD to CK results in a strongly quenched complex. (A) Normalized absorption spectra of the samples investigated (listed in the legend). The green thick lines indicate the central wavelength (±5 nm) of the excitation pulses used in the TA experiments presented in this work. (B) Raw (light color) and fitted (dark color) transient absorption kinetics of CK and CK-NTD at wavelengths indicative of the decay of the main bleach (∼675 nm) and rise of the quenching species (∼624 nm), measured with excitation centered at 694 nm (160 μW). The raw traces (in mOD) have been obtained by integrating the transient absorption signal of the experiments over an interval of ∼5 nm around the central wavelength. Fitted traces are a result of the model presented in the main manuscript in Figure 5. (C) Raw transient absorption spectra of CK and CK-NTD obtained by integrating over the time interval of 1 to 100 ps, corresponding to the rise of the signal in CK-NTD shown in (B).

The absorption spectra of the samples are shown in Figure 2A. The spectrum of CK-NTD shows a “baseline” that could be compatible with the presence of scattering particles in solution after the resuspension of the complex. It is important to mention, however, that the precipitation and resuspension did not affect the capacity of NTD to induce quenching in CK, as shown in Figure 1D (emission before and after precipitation, upon addition of NTD). The fact that the shape of the emission spectrum of CK was not modified by the addition of NTD indicates that the complex did not undergo aggregation.^28^

In the main manuscript we focus on the results of CK-NTD excited at 694 nm. The results upon 520 and 630 nm excitations are shown in the Supporting Information (Figure S7). In agreement with the fluorescence data, the raw TA data (Figure 2B) show that CK-NTD is strongly quenched: i.e. the bleach (≈675 nm) mostly decays with a time constant on the order of 10^2^ ps in CK-NTD, and of 10^3^ ps in CK. Notably, from the raw data it was found that in CK-NTD a signal centered at ≈625 nm rises in a few ps and decays on a time scale similar to the one of the ≈675 nm decay, whereas in CK the 625 nm band remains constant over such a large time scale and only decays on the order of 10^3^ ps. The population of an additional species in CK-NTD in the interval 1 to 100 ps, which is absent in CK, is also clear from the raw spectra integrated over the same interval (1 to 100 ps) in Figure 2C.

**Figure 3:**
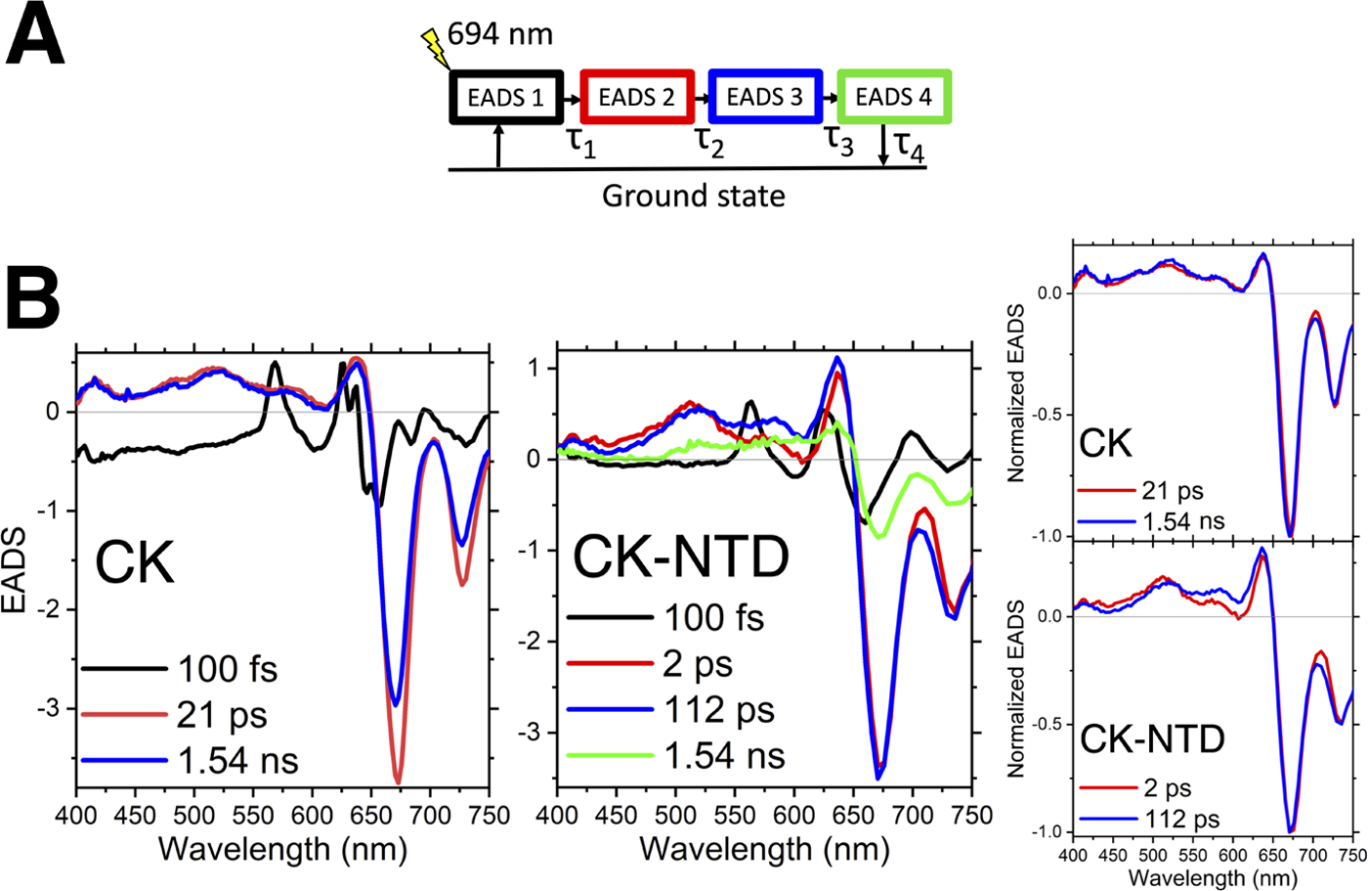
Different excitation energy transfer pathways are present in CK and CK-NTD. (A) Global sequential model applied to the raw data to retrieve the evolution associated difference spectra (EADS) reported in (B). (B) The two full sets of EADS for CK and CK-NTD are shown with the associated lifetimes. The first EADS of CK and CK-NTD have been multiplied by a factor of 0.2 for clarity. The normalized red and blue EADS from the model in (A) are reported on the right.

By applying a global sequential model (Figure 3A) to fit the raw TA data, we could satisfactorily describe the CK and CK-NTD data sets with 3 and 4 components, respectively. Fitting CK-NTD with 3 components resulted in a misfit, as shown in Figure S9. The evolution-associated difference spectra (EADS) obtained from this analysis are reported in Figure 3B. The first EADS corresponds to a subps time constant (100 fs) and contains contributions of coherent artifacts. The second EADS is attributed to excitation-energy transfer and equilibration between APC chromophores. This component is faster in CK-NTD than in CK (2 ps versus 21 ps), suggesting that a fast event might occur in this time window in the quenched sample. The 1.54 ns component represents the decay of the unquenched fraction of CK in both samples. In CK-NTD this component is minor, while the majority of the decay (≈76%) occurs in 112 ps. Such quenching yield and time scale were observed with all excitation powers used (Figure S1). Notably, analogous quenching yield and time scale (80% quenching in a few hundred ps) have been reported in vivo and in vitro in systems where the full OCP is active and bound to CK,^29−31^ indicating that NTD drives a quenching mechanism equivalent to the one driven by OCP^R^. This conclusion is supported by several additional results: (i) NTD alone is able to induce quenching on the whole PBS complex (CK plus rods);^32^ (ii) the structure of the isolated NTD is almost identical to that of NTD in the OCP-PBS quenched complex^15,24^; (iii) the same mutation in NTD and OCP inhibits quenching in PBS.^15^ This implies that NTD represents the minimal unit required to activate OCP-dependent NPQ in the core of the PBS in cyanobacteria.

As shown in Figure 3B, the second and third EADS of CK maintain overall the same spectral features: i.e. the combination of the ground state bleach and stimulated emission (≈675 nm) and the excited-state absorption (ESA) of the APC bilins (≈400–650 nm). Their only spectral difference consists in the blue shift of the bleach of the third EADS with respect to the second one, which is due to uphill energy transfer (excitation at 694 nm excites prevalently the most red species). The same blue shift is also observed in CK-NTD (Figure 3B). However, the third EADS of CK-NTD displays a distinct spectral change with respect to the second EADS also in the ≈400–650 nm region. Such a spectral change observed in the 2 ps component in CK-NTD is absent in CK. The spectral change is evident also in the raw data, as shown in Figure 2C.

The spectrum of the (mixture of) species that rises in 2 ps in CK-NTD can be determined via a model numerically equivalent to the sequential one, in which all the species decay in parallel (Figure S2A). The resulting spectra are called decay-associated difference spectra (DADS) and are presented in Figure S2B. The species involved in the spectral changes observed in 2 and 21 ps in CK-NTD and CK, respectively, are represented by the second DADS: the bandshift above ≈650 nm indicates energy equilibration taking place between APC680 and APC660 in both CK and CK-NTD, and is representative of the blue shift observed in the EADS due to uphill energy transfer. However, at variance with CK, the second DADS of CK-NTD shows a distinct, additional spectral feature which is mostly positive in the ≈400–530 nm range and negative in the ≈530–650 nm one. Such a feature is absent in CK (Figure S2B) and, given the spectrum, cannot be assigned to APC.

### Binding of NTD to CK Increases the Conformational Freedom of Canthaxanthin

To understand whether canthaxanthin is involved in the spectral evolution in the ≈400–650 range observed in 2 ps in CK-NTD (Figure 3B), we analyzed the excited-state dynamics of CK-NTD after carotenoid excitation (520 nm, Figure 2A). To aid the interpretation of the spectra and lifetimes of canthaxanthin in CK-NTD, TA experiments were also run on the isolated NTD. To limit bias (due to spectral selection^33−35^) in populating the excited states of canthaxanthin, two different excitation wavelengths were used on NTD (475 and 520 nm, Figure 2A).

To resolve the excited-state spectra of canthaxanthin in NTD unbound (NTD sample) or bound to CK (CK-NTD sample), we fitted our TA data with the compartmental models (target analysis) described in detail in the Supporting Methods (Supporting Information) and in Figures S3 and S7. The spectra obtained are called species-associated difference spectra (SADS).

**Figure 4:**
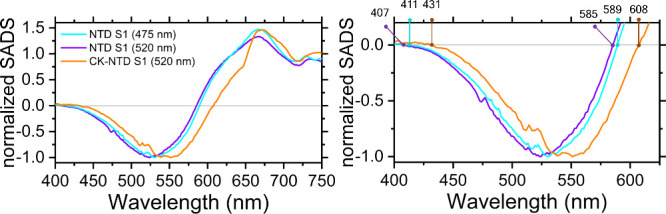
The S1 state of canthaxanthin is red-shifted in CK-NTD with respect to NTD. Left: normalized SADS assigned to the S1 state of canthaxanthin in NTD and CK-NTD, after excitation at 475 nm (NTD) and 520 nm (NTD and CK-NTD), as indicated in the legend. Right: a zoom of the spectra shown on the left, with indicated the zero-crossing points (in nm) used to compute the band shift of the S1 SADS of canthaxanthin in CK-NTD with respect to NTD.

By comparing the SADS of canthaxanthin in NTD (Figure S3) and CK-NTD (Figure S7), we obtained information on whether the binding of NTD to CK affects the carotenoid energetics. From the S1 spectra of NTD and CK-NTD (Figure 4) we found that the GSB of CK-NTD is significantly shifted toward lower energies. By comparing the zero-crossing points of the GSB of NTD and CK-NTD (Figure 4), we found that canthaxanthin undergoes a spectral shift of ≈21 nm toward the red, upon binding of NTD to CK.

A large red shift (40 nm) and a vibronic-less structure is also observed in the absorption spectrum of canthaxanthin when the carotenoid is bound to NTD/OCP^R^, with respect to OCP^O^. Such changes have been rationalized in terms of both structure and environment contributions via a multiscale atomistic approach.^36^ In ref (36) it was found that the conformational freedom of the terminal rings of canthaxanthin is higher in NTD than in OCP^O^. This allows canthaxanthin to populate more frequently conformers with a larger conjugation which, in turn, cause the redshift of the NTD absorption. However, it should be noted that, while conformational disorder has been shown to play a role in the red shift of isolated OCP,^36^ the change in the protein environment surrounding canthaxanthin in CK-NTD might also be responsible or contribute to it.^37^ Several charged residues of the protein subunits of CK called ApcA and ApcB have been found in the vicinity of canthaxanthin in the high-resolution structures of OCP bound to the PBS.^24,37^ These residues have been shown to have an impact on the electronic structure of canthaxanthin^37^ and might therefore contribute to or cause the red shift in absorption observed in Figure 4. Therefore, the fact that the spectrum of canthaxanthin is ≈21 nm more red-shifted in CK-NTD than in NTD, is indicative of canthaxanthin changing conformation and/or environment upon NTD binding to CK.

### NPQ Takes Place in the Core of the Phycobilisome via Excitation Energy Transfer to a Singlet Excited State of Canthaxanthin

**Figure 5:**
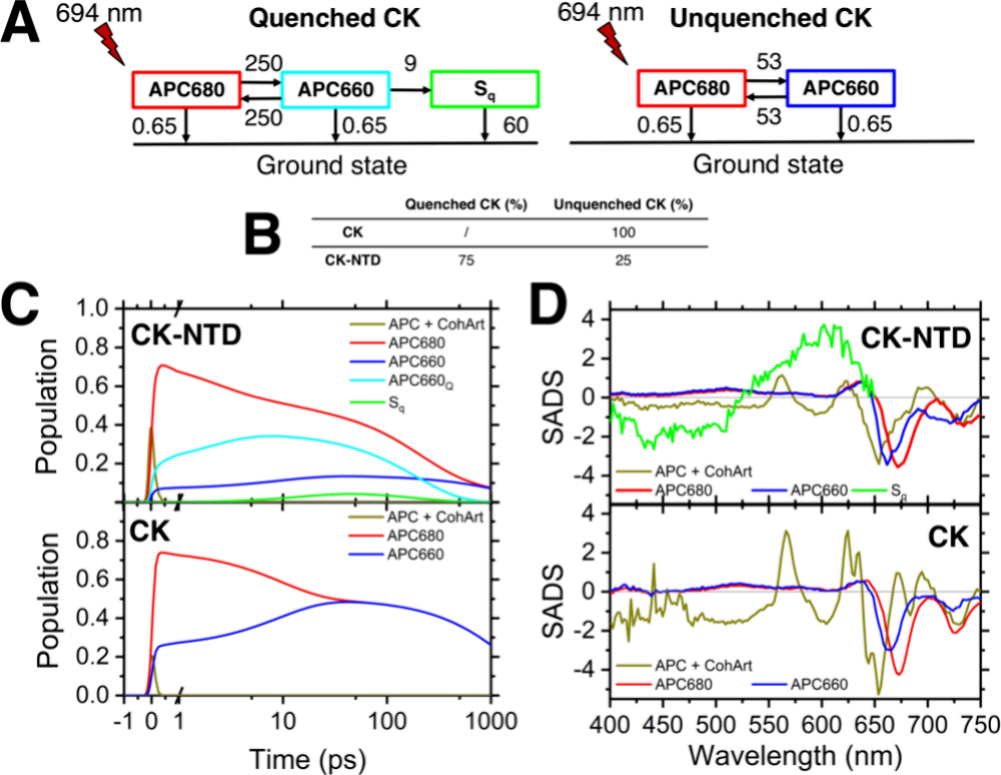
Compartmental model for the 694 nm excitation of CK and CK-NTD. (A) Heterogeneous compartmental model used to fit the TA data of CK and CK-NTD, obtained after excitation at 694 nm in annihilation-free conditions (40 μW). The red bolt indicates the species predominantly excited by the 694 nm excitation (APC680). The heterogeneous model consists of two megacomplexes to describe the excited-state dynamics of the quenched and unquenched fractions of CK. Arrows represent energy transfer processes and decays to the ground state. All rates are in ns^–1^. Kinetics faster than the IRF (<100 fs) were modeled via a precursor species (APC + CohArt) not shown in (A) for clarity. The complete model is explained in the Supporting Methods (Supporting Information) and in Figure S5. (B) Distribution (%) of the quenched and unquenched CK fractions populated by the initial excitation in the CK and CK-NTD samples. (C) Concentration profiles of all the species included in the compartmental model in (A). The quenched and unquenched APC660 species are labeled as APC660_Q_ and APC660, respectively. The maximum amplitude of the *S*_q_ concentration is 0.05. (D) SADS obtained from the model shown in (A).

To identify the spectrum of the quencher in CK-NTD, compartmental models were applied to fit all the TA data sets of CK and CK-NTD. The complete set of models and the results obtained at different excitation wavelengths (520, 630, and 694 nm) and powers are reported in the Supporting Information. Below we focus on the model applied to the TA data of CK and CK-NTD excited at 694 nm (Figure 5A–C). This low energy excitation (694 ± 5 nm) was chosen because of its marginal overlap with the absorption spectrum of CK (Figure 2A), which allowed us to excite selectively only a small pool of APC bilins (mainly APC680) and therefore drastically limit the possibility to incur in multiple excitations per single complex (that would result in singlet–singlet annihilation).

For the CK sample, a single homogeneous scheme that models the unquenched CK dynamics is sufficient to fit the TA data.

Below we focus on the model applied to the TA data of CK and CK-NTD excited at 694 nm (Figure 5A–C). This model is based on the following assumptions:

1. For the CK-NTD sample, the total population of excited species is modeled with two schemes, also defined as megacomplexes, describing the excited state dynamics of the quenched or the unquenched CK complexes (Figure 5A–C). This is a heterogeneous model where the relative amount of initial excitation assigned to the quenched/unquenched CK fractions (75/25) is based on the ≈76% quenching yield observed via TA (Figure 3B). For the CK sample, a single homogeneous scheme that models the unquenched CK dynamics is sufficient to fit the TA data. The scheme adopted for unquenched CK is identical in the two samples;
2. The pulse at 694 nm preferentially excites the lowest energy APC pigments (APC680) (Figures 5A and 2A). Then, APC680 undergoes “uphill” energy equilibration with APC660 and both species decay to the ground state in ≈1.5 ns. This lifetime of unquenched APC bilins matches our results (Figure 3B) and previous ones from time-resolved spectroscopy on CK.^30,38^
3. The model describing the quenched fraction of CK-NTD is identical to that of the unquenched CK, but contains an additional energy transfer pathway (Figure 5A). Specifically, APC660 (in equilibrium with APC680) transfers excitation in 111 ps to an unidentified state, here called *S*_q_. *S*_q_ is a dissipative channel that decays back to the ground state in 17 ps.

Full details of the schemes and rates used for this analysis are provided in the Supporting Information. In Figures S6 and S8, it is shown that this model provides an excellent fit for our TA data.

This model agrees with the conclusions of several in vitro and in vivo fluorescence studies,^30,39^ which identify APC660 as the site of NPQ in cyanobacteria. This conclusion is further supported by the recent OCP-PBS structure,^24^ which shows NTD interacting more closely with the CK ApcA/ApcB bilins that emit at 660 nm (APC660). Additionally, our models provide experimental evidence that the OCP-related quenching of PBSs follows an inverted kinetics regime–i.e. a slow transfer (111 ps) to a fast decaying quencher (17 ps) (Figure 5A) – similarly to what was previously found in a variety of photosynthetic light-harvesting complexes of plants,^40−43^ mosses^44^ and algae,^45^ and proposed based on the recent OCP-PBS structure.^24^

What is the quencher in cyanobacteria? The SADS of the quencher *S*_q_, is presented in Figure 5D and shows a bleach between ≈400–530 nm and a positive ESA between ≈530–650 nm. This is the region where we observed a spectral change in the raw data (Figure 2B,C) and via global analysis (Figures 3B and S2B). Importantly, the spectral features characteristic of *S*_q_ in the whole ≈400–650 nm range are absent in the SADS of both APC660 and APC680 and can thus not be assigned to them.

**Figure 6:**
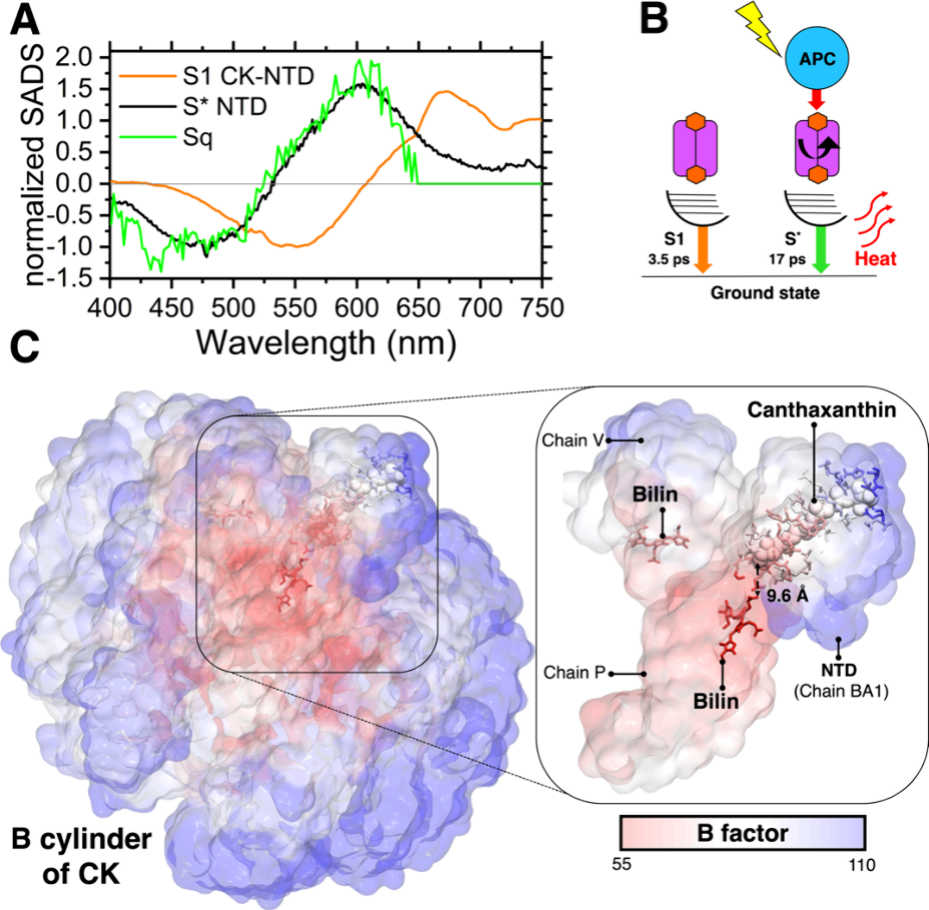
The quencher in CK-NTD is a carotenoid singlet excited state. (A) The SADS of the *S*_q_ state (*S*_q_ of CK-NTD after excitation at 694 nm, Figure 5D) is reported together with the SADS of canthaxanthin S1 in CK-NTD (S1 of CK-NTD after excitation at 520 nm, Figure S7) and S* in NTD (S* of NTD after excitation at 475 nm, Figure S3). The SADS are normalized SADS, allowing comparison of the spectra. (B) A simplified model of the energetic and conformational landscape of canthaxanthin in CK-NTD is shown. The presence of different singlet excited states (S1 and S* in this case) implies that the carotenoid is set in different conformations in CK-NTD, as indicated by a black curved arrow in the Figure. The quencher of APC (*S*_q_ state, Figure 5D) is here assigned to S*, due to the striking similarity in spectra and lifetime. (C) Structure of the basal cylinder of CK with NTD bound, from the resolved structure of the quenched OCP-PBS complex (PDB 7SCB,^24^). The pigments and the protein surfaces, which are shown for the whole cylinder in the main picture and for selected chains in the inset, are colored according to the B factor reported in the resolved structure.^24^ A high B factor may depend on increased thermal motion of the atoms and heterogeneity in structure, among other factors.^58^ In the inset, the two nearest bilins to canthaxanthin are shown as sticks and the carotenoid itself as van der Waals spheres. The distance between the two nearest atoms of the bilin and canthaxanthin is also indicated.

Can the spectrum be assigned to canthaxanthin? This possibility is explored in Figure 6. *S*_q_ does not correspond to the S1 SADS retrieved for canthaxanthin in CK-NTD (Figure S7), which is significantly red-shifted as compared to *S*_q_ (≈83 nm, as measured from the zero-crossing points). However, the spectrum matches well with that of another dark state called S* (Figure 6A). This state has been identified in canthaxanthin in solvents of different polarity,^46,47^ in OCP,^34,48^ NTD (Figure S3), NTD homologues,^33,34^ and in hECN binding OCP.^16^ While its origin remains controversial, several independent studies have assigned it to a singlet excited state associated with a distinct conformer of the carotenoid, populated via a distortion of the conjugated chain.^16,34,40,49,50^ In agreement with this proposal, S* has been found to be functional both as an excitation energy donor and as an acceptor in a variety of natural and artificial antenna systems.^40,43,51,52^ Other studies have assigned the origin of the S* spectral feature to a hot ground state, instead.^53,54^ The S* spectrum of canthaxanthin shows a bleach in the 400–520 nm region and displays an ESA starting from ≈520 nm toward longer wavelengths (Figure S3). Strikingly, this corresponds to the region in which the bleach and ESA of *S*_q_ lie and, indeed, the spectra of S* and *S*_q_ match to a great extent (Figure 6A). The S* lifetime of canthaxanthin was reported to be dependent on the excitation wavelength^33,47^ and to be consistently longer than that of S1, in all environments and at all excitation wavelengths.^33,46,47,55^ This is also the case in our NTD experiments, which show that the lifetime of S* is longer than that of S1 (17 ps vs 3.5 ps, respectively) (Figure S3). By assigning the lifetime of S* to *S*_q_, an excellent fit of the TA data of CK-NTD was obtained (Figures S6 and S8). Given the striking similarity in spectra and decay rates, we conclude that a singlet excited state of canthaxanthin with the characteristics of S* is responsible of quenching CK, when NTD is bound to it (Figure 6A,B). The distortion of canthaxanthin might explain the acquisition of dipole strength in the *S*_q_ ← S_0_ transition,^40,43,50^ and therefore the enhanced coupling between the carotenoid and the APC pigments, which could then favor excitation energy transfer from the bilins to the carotenoid. The resolved structure of the quenched OCP-PBS complex^24^ is compatible with our results: the larger B factor (or temperature factor) of canthaxanthin and of its binding pocket with respect to the one reported for APC bilins suggests that the carotenoid is characterized by molecular motion and heterogeneity in structure with respect to the more rigid bilins (Figure 6C). The conformational freedom of canthaxanthin in CK-NTD may allow the carotenoid to adopt a subset of ground state conformations from which S* can be populated via excitation energy transfer from the bilins.

It must be noted that the resolved structure of the quenched OCP-PBS complex^24^ shows that a connected PB system is quenched by two OCP dimers bound to two different parts of the core. This suggests that either multiple NTDs bind to the core in CK-NTD without the need of the C-terminus (a possibility that cannot be excluded based on the resolved structure^24^), or that the availability of a single quencher in a connected system is sufficient to trigger quenching, without the need of three additional OCPs.

## Conclusions

In this work we assembled in vitro a functional complex in which the carotenoid-binding active domain of OCP, NTD, is bound to the putative quenching site of the phycobilisome–the core. We showed that this minimal system triggers a quenching mechanism with efficiency and rate identical to the ones of the quenching activated by the full OCP on the phycobilisome core in vitro and in vivo. Our results indicate that when NTD binds to CK, canthaxanthin is subjected to an enhanced conformational disorder of its terminal rings and/or a change of environment, as supported by the red shift of its absorption (+ ≈21 nm). In CK-NTD, activation of quenching in CK is enabled via a structural switch of the carotenoid: more in detail, we showed that quenching takes place via excitation energy transfer from the singlet excited state of APC phycobilins to a singlet excited state of canthaxanthin. Canthaxanthin accesses this state by changing structure. To date, CK-NTD represents both the largest quenched system (72 bilin pigments per core plus one ketocarotenoid per NTD) and the first one composed of two distinct subunits (CK plus NTD), in which the dissipative mechanism has been identified. Because an equivalent mechanism of quenching: i.e. excitation energy transfer to a carotenoid singlet excited state, has been recently demonstrated in plants,^40−43^ algae^45^ and mosses,^44^ and in cyanobacterial high light-inducible proteins,^59^ our results suggest the presence of a common strategy for photoprotection in oxygenic photosynthesis.

## Methods

### Sample Isolation

Recombinant holo-NTD was produced in canthaxanthin-producing *E. coli* BL21 (DE3). The expression method used to obtain the holo-proteins was described previously.^12^ The CK complex was purified from a *Synechocystis* PCC 6803 mutant, lacking the PBS rods, as described elsewhere.^21^ The CK-NTD complex was prepared by mixing CK at 0.05 μM, NTD 1 μM (20 per CK) in phosphate buffer 1.2 M (final volume was 5 mL) at RT for 10 min. The formation of the complex was followed by measuring the decrease of CK fluorescence before and after the addition of NTD using excitation at 600 nm. To separate the complex from the free NTD, CK-NTD was precipitated by ultracentrifugation at 48,000 rpm at 23 °C for 3 h. Then, the CK-NTD complex was resuspended in phosphate buffer 1.2 M to a final optical density of 4.5 at 630 nm.

### Steady-State Spectroscopy

Absorption spectra were acquired at room temperature on a Varian Cary 4000 UV–vis spectrophotometer. Fluorescence emission spectra were acquired in a CARY Eclipse spectrophotometer (Varian) at room temperature with an excitation centered at 600 nm. The sample was diluted to an OD of 0.036 and placed in a 1 cm cuvette and stirred.

### Ultrafast Spectroscopy Experiments and Analyses

TA measurements were run on the setup described in ref (16) with some modifications. Amplified mode-locked pulses centered at ≈800 nm were generated at 1 kHz repetition rate by a Ti:sapphire Libra system (Coherent) and splitted (80/20) in pump and probe paths, respectively. Depending on the experiment, the pump wavelength was tuned to 475, 520, 630, 694 nm, via optical parametric amplification in an OperA SOLO system (Coherent), and further reduced to 10 nm fwhm using interference filters (THORLABS). The time delay was controlled via an optical delay line up to ≈1 ns, by delaying the pump. The probe white-light continuum was generated by focusing the 800 nm-pulse in a CaF_2_ plate, mounted on a home-built rotating stage to avoid damage. The probe was dispersed via a prism spectrograph on a 1024-pixels back-thinned FFT-CCD detector (S7030–1006, Hamamatsu). Pump and probe polarizations were set at magic angle (54.7°). Data were collected on a shot-to-shot basis, and the pump and probe pulses were modulated at frequencies of 500 and 250 Hz, respectively, using mechanical choppers (THORLABS). The optical density of the samples was set to ≈4 cm^–1^ in the case of CK and NTD and ≈2 cm^–1^ in the case of CK-NTD. The sample was kept at room temperature in a 2 mm quartz cuvette and refreshed via a home-built shaker throughout the measurement. Sample integrity was checked by inspecting the signal stability over multiple scans. Experiments with different excitation powers were conducted on both CK and CK-NTD to obtain data sets with an increasing signal-to-noise ratio. In all cases we were able to completely or largely prevent singlet–singlet annihilation effects, depending on the case, as shown in Figure S4 and explained in the Supporting Methods in the Supporting Information. These power-dependent experiments allowed us not only to quantify the amount of singlet–singlet annihilation, but also to produce a compartmental model (target analysis) where power dependency, when present, is accounted for (Supporting Methods in the Supporting Information). Multiple scans were acquired at each experiment and averaged before global or target analysis. In addition, we averaged the data to obtain a final wavelength step of 2 nm. Global and target analyses were applied to the TA data following the principles reported in ref (56) and explained in detail in the Results section. The instrument response function (IRF) of the experiments was estimated from the fitting to be ≈160 fs fwhm. The ultrafast time constants (≪100 fs) and the ones slower than 1 ns were fixed in the global analyses, due to the time resolution of the experiments. In each analysis, the chirp of the supercontinuum probe was corrected for via a parametric description of the IRF.

## Data Availability

All data used in this work are presented in the main text and Supporting Information and there is no restriction on data availability.

## Supporting Information Available

Detailed compartmental model, additional results, and analyses of transient absorption experiments (PDF)
