# Supplementary material for: The Orange Carotenoid Protein Triggers Cyanobacterial Photoprotection by Quenching Bilins via a Structural Switch of Its Carotenoid
Source: J Am Chem Soc. 2024 Jul 26;146(31):21913–21. doi: 10.1021/jacs.4c06695 (PMC11311238; doi:10.1021/jacs.4c06695)
Supplement: Supplementary file 1 — ja4c06695_si_001.pdf [file ja4c06695_si_001.pdf]

The orange carotenoid protein triggers cyanobacterial photoprotection by quenching bilins via a structural switch of its carotenoid

Nicoletta Liguori[1,], Ivo H.M. van Stokkum[1], Fernando Muzzopappa[2], John T. M. Kennis[1], Diana Kirilovsky[2], Roberta Croce[1,*]*

[1]Department of Physics and Astronomy and Institute for Lasers, Life and Biophotonics, Faculty of Sciences, Vrije Universiteit Amsterdam, de Boelelaan 1081, 1081 HV Amsterdam, The Netherlands;
[2]Institute for Integrative Biology of the Cell (I2BC), CNRS, CEA, Université Paris-Sud, Université Paris-Saclay, Gif sur Yvette, 91198, France

Supporting methods: compartmental models.

Compartmental models (target analysis) were used to fit the TA datasets obtained upon 694 nm excitation for CK and 630, 520 and 694 nm excitations for CK-NTD. The main features of the compartmental models for the 694 nm excitation are reported in the main text and are here explained more in detail, together with the models applied to the other datasets. To help the reader, we first present the simplest model used in this work: *i.e.* the compartmental model describing the CK sample measured with the most selective excitation (694 nm) and with the lowest excitation power. This model represents the base for the models that include quenching (in the case of CK-NTD) or power-dependent kinetics. All the other models are then introduced step-by-step in the following sections.
The target model used for the TA data of NTD after 475 and 520 nm excitations is shown in **Fig. S3**.

694 nm excitation.

The CK sample. The TA data of the CK sample are fully described by a single scheme (called *Unquenched CK*) containing two compartments of unquenched APC pigments: *i.e.* APC680 and APC660 (**Fig. 5.A, S5.A**). During and immediately after excitation at 694 nm a precursor species is formed, with a lifetime of ≈100 fs. The SADS of this precursor (APC + CohArt SADS, **Fig. 5D, S5.B**) shows multiple positive and negative bands that we tentatively assigned to a combination of excited-state absorption (ESA), Raman scattering signal and coherent artifact. From this precursor, 75% and 25% of the excitations are transferred to the APC680 and APC660 compartments, respectively. The fraction of quenched/unquenched population explained later in the model for the CK-NTD sample is unrelated to this. The equilibration time between APC680 and APC660 (19 ps) is expected to be a weighted average of the intradisk, interdisk and intercylinder equilibration timescales[1,2]. It must be noted that the population is also a consequence of entropy. In this case there are 66 APC660 pigments and 6 APC680 pigments. Thus, the enthalpic advantage of the APC680 is balanced by the entropic advantage of the APC660, resulting in a quasi-equilibrium where both populations are equal[1]. Both APC680 and APC660 decay to the ground state in ≈1.5 ns. This lifetime matches the lifetime of unquenched APC bilins previously measured via time-resolved spectroscopy on CK[1,2] and agrees with our global analyses results (**Fig. 3.B**).
The CK-NTD sample. To model the TA data of the CK-NTD sample, an additional megacomplex is required. This megacomplex accounts for the large fraction of quenched CK complexes which, together with a smaller fraction of unquenched CK, is present in the sample. The megacomplexes are called *Quenched CK* and *Unquenched CK*, respectively. The *Unquenched CK* megacomplex is identical for both CK and CK-

NTD samples. The relative amount of initial excitation assigned to each scheme (75/25, respectively) has been chosen based on the amplitude of the quenched component measured via TA (76%, **Fig. 3.B**). The *Quenched CK* presents an additional compartment with respect to the *Unquenched CK*. This compartment is assigned to an unidentified species, called S_q. APC660 (which is in equilibrium with APC680) transfers excitation energy to this compartment in 111 ps. S_q decays back to the ground state in 17 ps, quickly dissipating the excitation energy of the *Quenched CK* complexes. The transfer rate from APC660 to S_q has been estimated from the data and agrees with the quenched lifetime component of a few hundred ps measured in previous, independent time-resolved spectroscopy studies on CK[3,4]. Different decay rates have been tested for the quencher S_q, and rates in the range of 60±10 ns^{-1} all resulted in excellent fits with similar RMS error. Given the similarity in spectrum between S_q and the S* state of NTD (independently estimated, **Fig. S3**), we have then fixed the S_q decay rate to 60 ns^{-1}.

Power dependent study. These simple models fully describe the TA data of CK and CK-NTD obtained at the lowest power (40 μW). To obtain also datasets with a higher signal-to-noise ratio, we additionally measured CK and CK-NTD at higher powers. However, at higher powers, singlet-singlet annihilation might contribute to the excited-state dynamics. To understand at which power such contribution should be taken into account in the model, additional independent measurements were run on CK at 694 nm excitation, with a power of 100, 160 and 320 μW. In all cases, the very low absorption cross-section of CK at 694 nm (**Fig. 2.A**), allowed to maintain a significantly low excitation density. Indeed, as in Ref. [5], the excitation density was computed as $\frac{N_{APC} \cdot \Delta OD_{max}}{2OD_{max}}$, where OD_{max} refers to the Qy region and N_{APC} is the number of APC per single CK. In CK the excitation density ranged between 0.5% (40 μW) and 6.8% (320 μW). Similarly, a power-dependent study with excitation at 694 nm was also conducted on CK-NTD and the excitation densities were estimated to range between 0.96% (40 μW) and 3.2% (160 μW). The time traces of CK and CK-NTD at the different powers are reported in **Fig. S4**. The excitation density is expected to be smaller than these estimates because, for these calculations, the totality of 72 APC pigments was considered to absorb at 694 nm, which is a large overestimation. Only a negligible number of APC pigments is instead expected to absorb significantly at this wavelength (mostly APC680).

These estimates suggest that the contribution of annihilation in all cases was maintained to null or limited values. To properly account for possible annihilation, we applied a modified scheme to the model presented in **Fig. 5.A** to each CK dataset. In the modified model (**Fig. S5.B)** an additional megacomplex, called *Unquenched CK with annihilation*, is added to the *Unquenched CK* one, in which APC680 decays at a faster rate (25 ns^{-1}). The fraction of CK belonging to the *Unquenched CK with annihilation* scheme at each power is reported in **Fig. S5.C**. This power-dependent model (**Fig. S5.A**) results in remarkably similar SADS amongst all the different powers used (**Fig. S5.B**).

In the case of the CK-NTD sample, two megacomplexes describing the *Unquenched CK with annihilation* and *Quenched CK with annihilation* fractions were added to the model in **Fig. 5.A**, each containing a faster decay of APC680 (25 ns^{-1}). From this model we estimated a contribution of annihilation equal to 0% at 40 μW and 14% at 160 μW.

Spectral assumptions. To limit the number of free parameters, a few spectral assumptions were made, similarly to what previously done in Ref.[5,6]: 1) the SADS of APC680 and APC660 were found to be similar below 649 nm (the ESA region) in CK

(**Fig. 5.B**). Therefore, in the target analysis of CK-NTD, the SADS of APC680 and APC660 are guided to be similar (but not identical) below 649 nm (the ESA region); 2) the SADS and decay rate of S_q were linked at 40 and 160 μW in CK-NTD. The SADS of S_q was forced to be zero above 649 nm, to avoid an imprecise estimation of its spectrum in the region were the spectral evolution of the APC bands takes place. For a reference on guidance spectra see Ref. [7].
This model resulted in an excellent fit, as can be assessed from the overlap of fitted and measured kinetics across the spectrum (**Fig. S6**).

630 and 520 nm excitations.
In line with the model developed for the 694 nm excitation, compartmental models consisting of *Unquenched* and *Quenched CK* megacomplexes were also used for the TA datasets of the CK-NTD sample, after 630 and 520 nm excitations. The only difference is that both *Unquenched* and *Quenched CK* schemes now contain three APC660 compartments (630 and 520 nm excitations), instead of one (694 nm excitation). $APC660_A$, $APC660_B$, $APC660_C$ represents the top cylinder, APC660 disks in the basal cylinders, and the APC660 in the mixed APC660/APC680 disks, respectively. The equilibration rates chosen for these models were based on the ones previously validated on CK for excitations also selective towards APC660, via independent time-resolved spectroscopy experiments[1] and represent intradisk, interdisk and intercylinder equilibration.
The models used for the 630 and 520 nm excitations are presented in **Fig. S7** and the datasets were fitted simultaneously: a precursor is instantaneously excited. This precursor decays in 0.19 ps and transfers the excitation energy to the APC660 compartments. The three APC660 compartments ($APC660_A$, $APC660_B$, $APC660_C$, **Fig. S7**) equilibrate amongst themselves and with APC680, and they all decay with the unquenched APC rate of 0.65 ns^{-1}. The SADS of the APC660 and APC680 species obtained via these models have their bleaches centered at ≈660 nm and ≈680 nm, respectively, as it should be expected and in agreement with the results for the 694 nm excitation (**Fig. 5.B**). Again, similarly to the model applied to the 694 nm excitation of CK-NTD (**Fig. 5.A**), in the *Quenched CK* pool, excitation energy is transferred from the APC660 compartments to S_q with rates of 5.6 and 8.9 ns^{-1}. Then, as before, S_q quickly decays to the ground state in 17 ps.
With the 520 nm excitation, NTD is directly excited in addition to CK (**Fig. 2.A**). The direct excitation of NTD was modeled by adding a megacomplex, called *Canthaxanthin*, which describes the excited-state dynamics of the carotenoid (**Fig. S7**). The *Canthaxanthin* megacomplex is identical to the one used to model the TA data of the NTD sample after 520 excitation, but it misses the compartment assigned to the S1' species resolved in the case of NTD (**Fig. S3**). No precursor in the canthaxanthin megacomplex was necessary to be included in the 520 nm excitation models of CK-NTD and NTD.
Spectral assumptions. Similar to the case of the 694 nm excitation (see above), the SADS of S_q is assumed to be zero above 649 nm, and the SADS of APC680 and APC660 are here assumed to be equal in the ESA region. The S_q SADS was guided by the SADS estimated with the 694 nm excitation. The SADS of APC660, APC680 and the NTD S_q have been linked between the two experiments at 630 and 520 nm excitations (**Fig. S7**). This model resulted in an excellent fit, as can be assessed in **Fig. S8**.

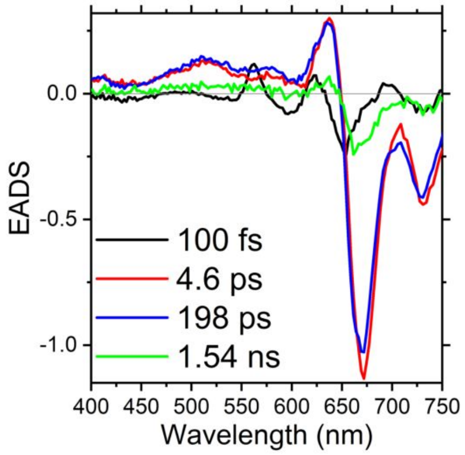

Figure S1. Sequential model applied to CK-NTD excited at 694 nm (40 uW). Evolution Associated Difference Spectra (EADS) obtained fitting the transient absorption (TA) experiments on CK-NTD, upon excitation at 694 nm with the lowest power (40 uW). The first EADS has been multiplied by a factor of 0.2 for clarity.

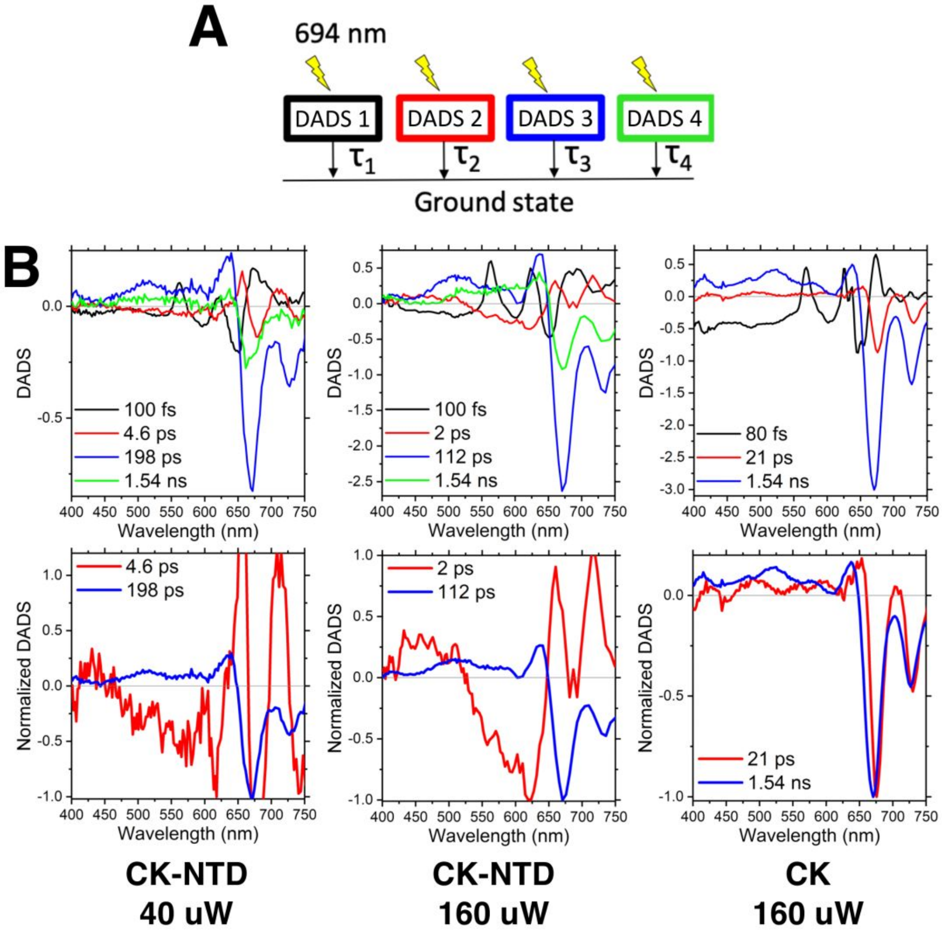

Figure S2. Parallel model for the 694 nm excitation of CK and CK-NTD. A. Scheme of the parallel model applied to obtain the decay associated difference spectra (DADS) shown in **B. B.** The DADS of CK-NTD (at 40 and 160 uW excitation power) and CK (160 uW excitation power) are shown above. Normalized DADS are shown on the bottom for selected components. The samples and excitation power used in each dataset are indicated below the plots. The first DADS have been multiplied by a factor of 0.2 for clarity.

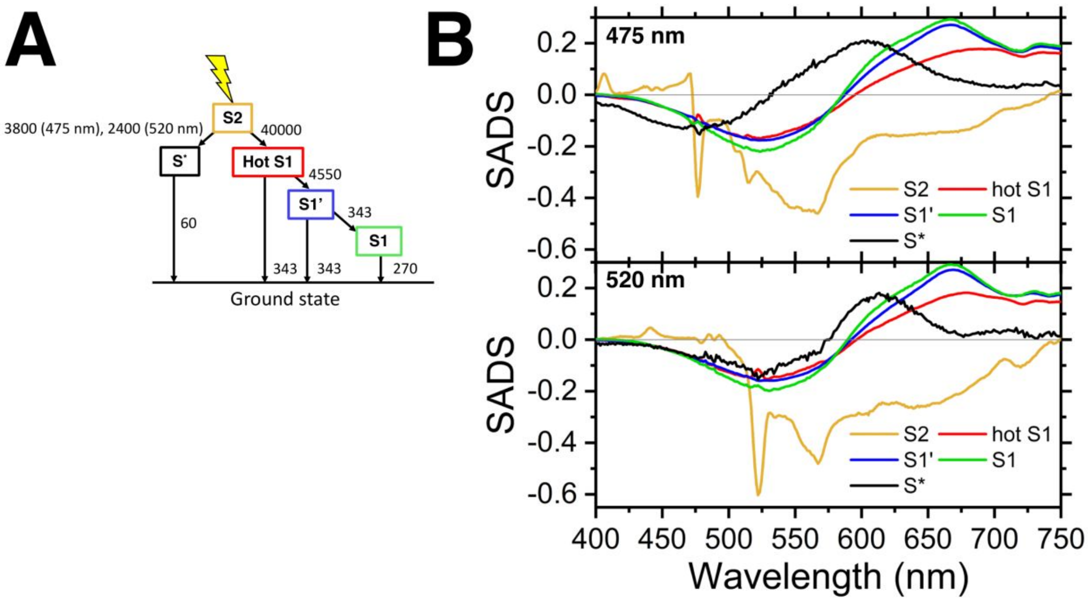

Figure S3. Compartmental model for the 475 and 520 nm excitations of NTD.
A. Compartmental model used to fit the TA data of NTD upon excitation at 475 and 520 nm. The yellow bolt indicates that S2 is the first state directly populated following each excitation. The models and rates used to fit the datasets obtained with the 475 and 520 nm excitations are identical, except for the rates of internal conversion from S2 to S^* which are estimated independently and are different (as indicated in the scheme). The higher rate at 475 nm excitation agrees with the expected higher probability of S* to be populated in NTD with respect to lower excitation energies (photoselectivity[8,9]). All rates are in ns^{-1}. SADS obtained from the model in **(A)**, at the excitation wavelength indicated on the plot. It must be noted that for this model a compartment called S1', because of the similarity in spectrum and lifetime with S1, was required to obtain an excellent fit of the TA data. After vibrational relaxation from hot S1 to S1', 50% of S1' decays directly to the GS, whereas the remainder relaxes to S1, which in turn decays to the GS in ~4 ps.

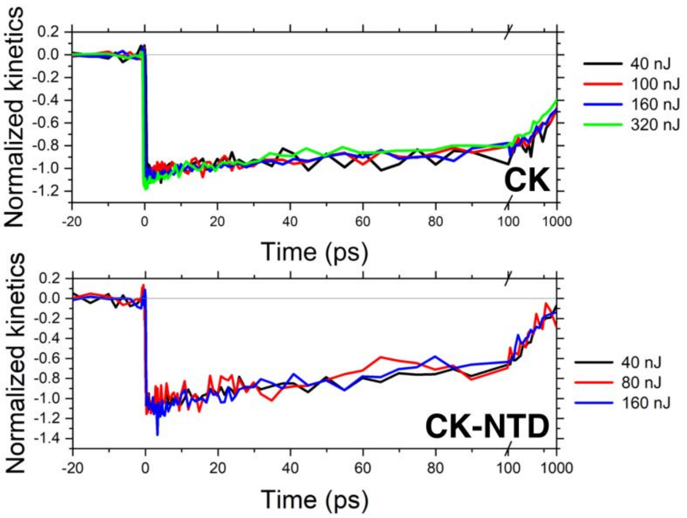

Figure S4. Power-dependent kinetics of CK and CK-NTD. Normalized traces (raw data) at the minimum of the ground state bleach of CK and CK-NTD, obtained independently after excitation at 694 nm, with different energies per pulse (indicated in the legend).

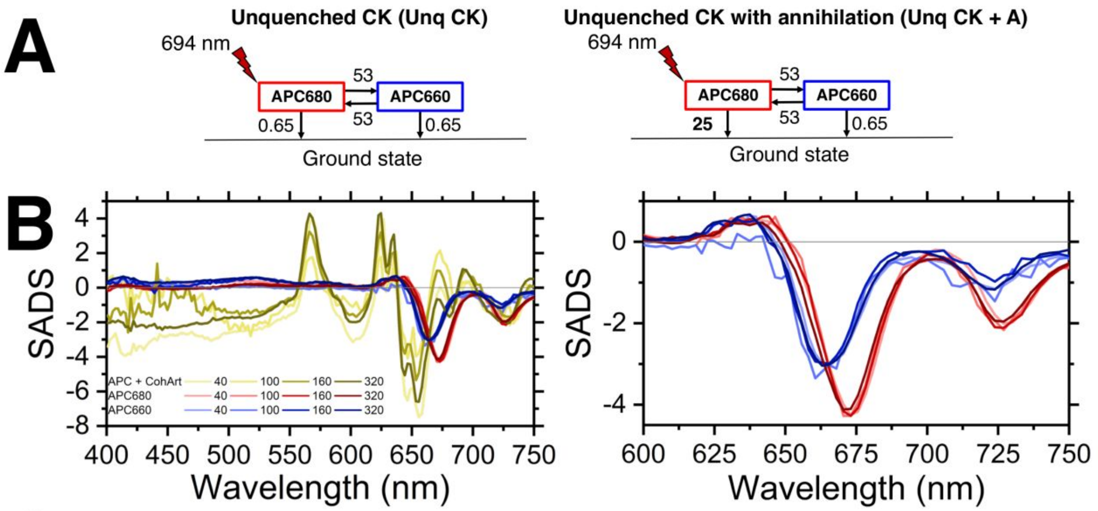

C

	40 uW	100 uW	160 uW	320 uW
**Unq CK**	100	91	86	78
**Unq CK + A**	0	9	14	22

Figure S5. Compartmental model for the 694 nm excitation of CK at different powers. A. Compartmental model used to fit the TA data of CK upon excitation at 694 nm at different excitation powers. The precursor (APC+CohArt, see Supporting Methods) is not shown for clarity. All rates are in ns^{-1}. **B.** Complete SADS (left) and a zoom of the bleach region (right), obtained from the model shown in **(A)**. **C.** Relative amount (%) of initial excitation assigned to the *Unquenched CK* (Unq CK) and *Unquenched CK with annihilation* (Unq CK + A) megacomplexes **(A)** at the different excitation powers used, as indicated in the table.

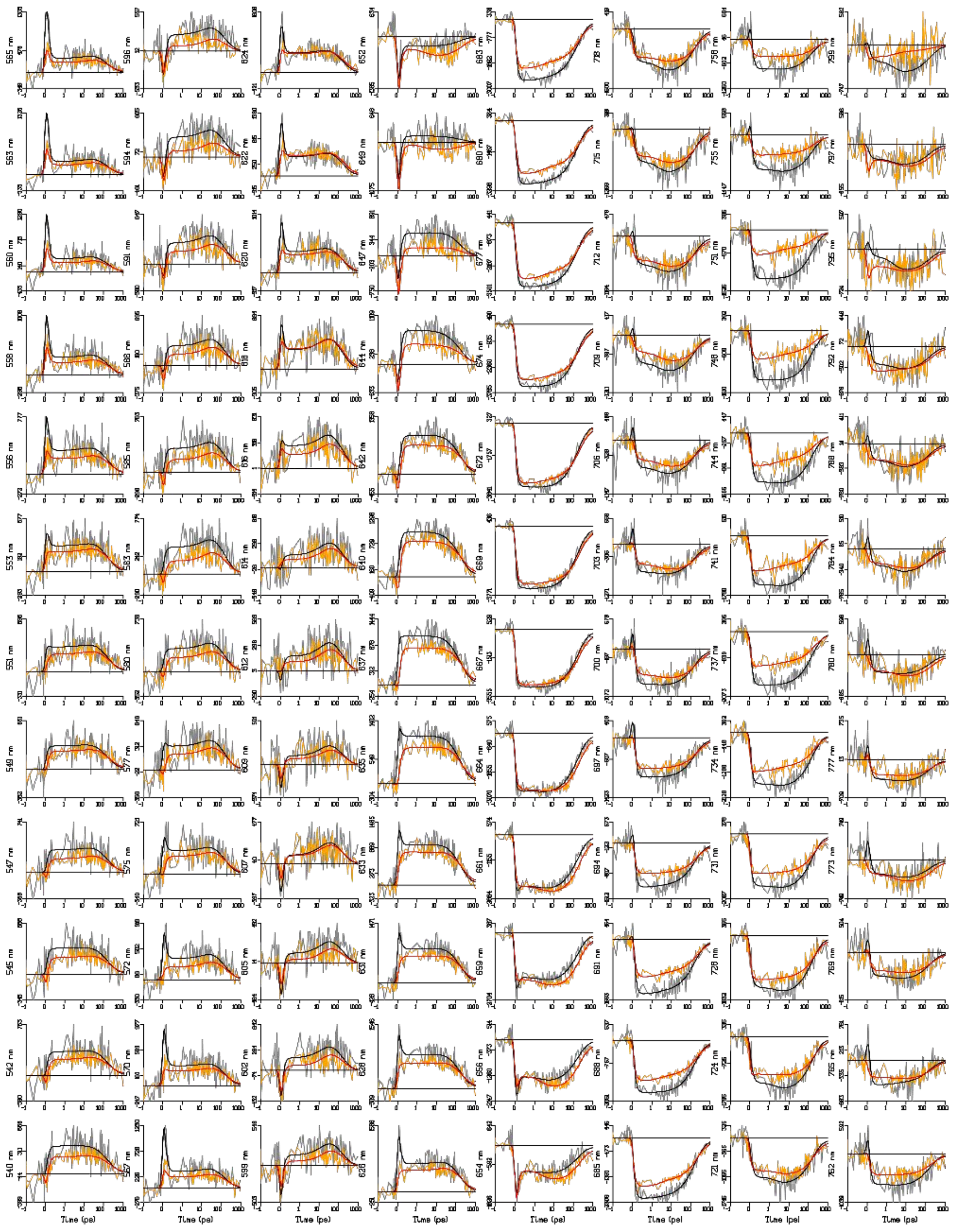

Figure S6. Transient difference absorption of CK-NTD at 96 wavelengths (indicated in the ordinate label) after 694 nm excitation. Color code: 160 uW (grey), 40 uW (orange). Black and red lines indicate the simultaneous target analysis fit. Note that the time axis is linear until 1 ps and logarithmic thereafter. Note also that each panel is scaled to its maximum. Overall root-mean square error of the fit is 107.7 μOD.

A

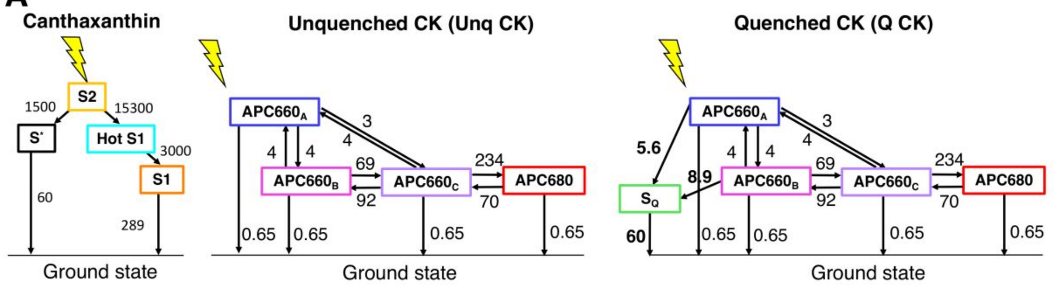

B

	630 nm	520 nm
Unq CK	14	6.5
Q CK	76	38.5
Annhilated CK	10	5
CX	0	50

C

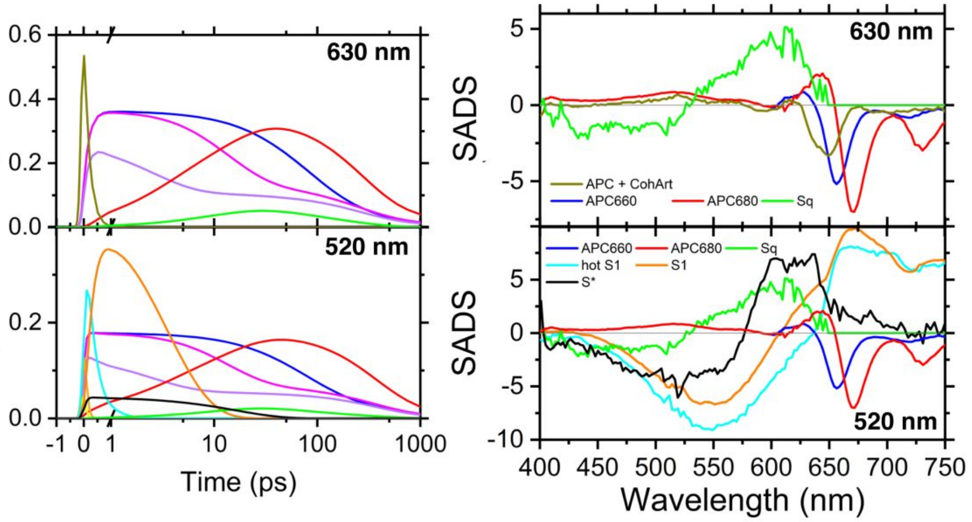

Fig. S7. Complete compartmental analysis on the TA data after 630 and 520 nm excitations of CK-NTD. A. Compartmental model used to fit the TA data of CK-NTD upon excitations at 630 and 520 nm. The instantaneous precursor (APC + CohArt) modeled in the 630 nm and 520 nm data (see Supporting Methods) is not shown for clarity. All rates are in ns^{-1}. **B** Relative amount (%) of initial excitation assigned to the *Unquenched CK* (Unq CK), Q*uenched CK* (Q CK) and *Canthaxanthin* (CX) megacomplexes **(A)**, at the different excitation wavelengths used. In both datasets a small fraction of annihilated *Unquenched* and *Quenched CK* was accounted for (10% and 5%, respectively), by setting the $APC660_A$ decays to 14 ns^{-1}. **C.** Concentration profiles (left) and complete SADS (right) obtained from the model shown in **(A)**. The excitation wavelengths to which each plot refers to are indicated on top of the figures. Note that the SADS of APC660, APC680 and the NTD S_q have been linked between the two experiments at 630 and 520 nm excitations and are therefore identical. The SADS of S2 and APC + CohArt (light orange and yellow concentration profiles on the left) are not shown for clarity. In **Figure S3**, the S* SADS after 475 and 520 nm excitations of NTD differ, which is attributed to photoselectivity[8,9]. The S* SADS in **Figure S7.C**, bottom right, resembles the S* after 520 nm excitation of NTD. The Sq (in CK-NTD) resembles the S* after 475 nm excitation of NTD. The intensity increase of S1 near 650 nm in **Fig. 6.A** is unrelated to the Sq SADS being forced to zero. The dynamics of these states are different as can be seen from the populations in **Fig. S7.C**.

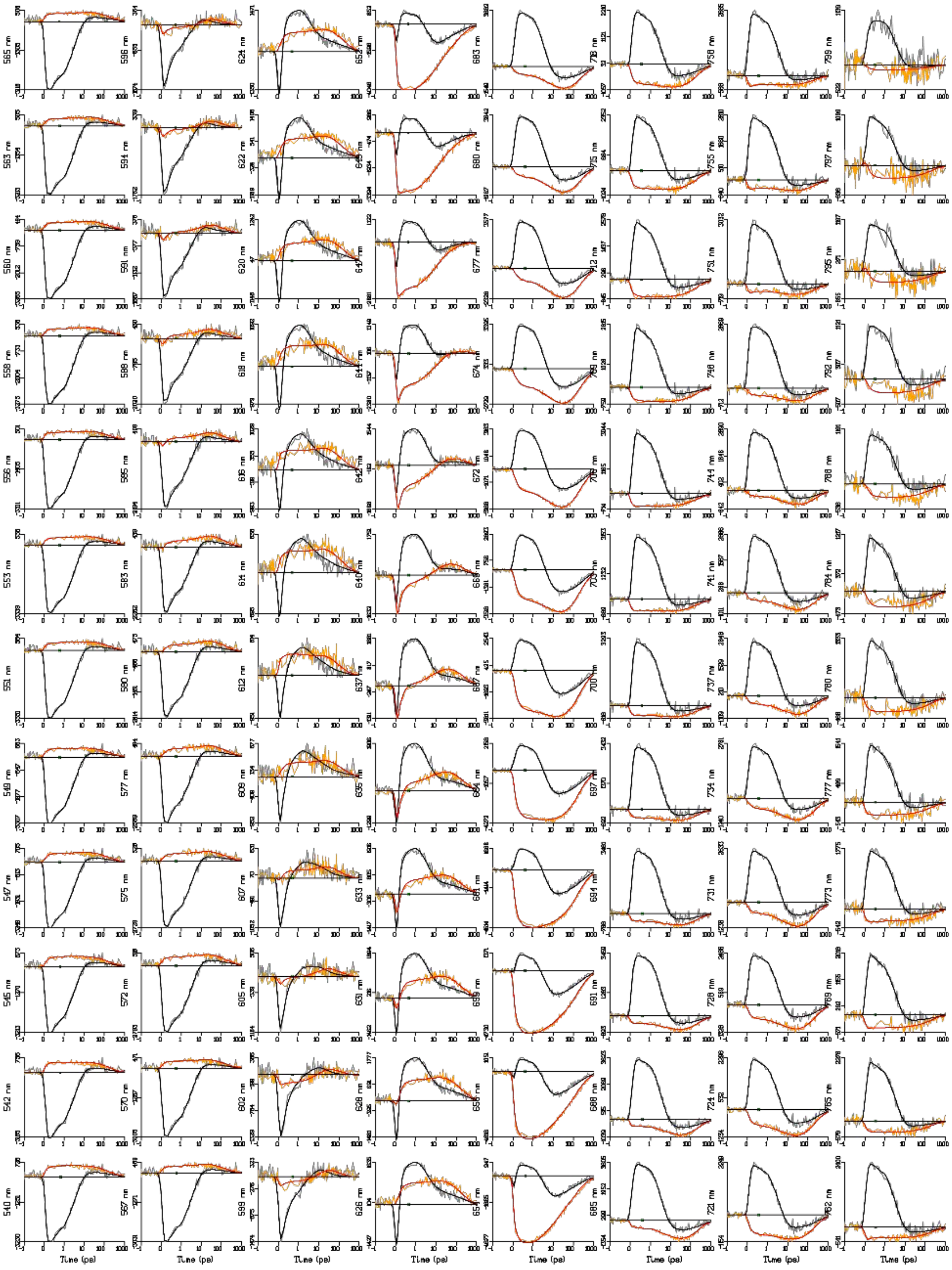

Figure S8. Transient difference absorption of CK-NTD at 96 wavelengths after 520 and 630 nm excitations. Color code: CK-NTD after 520 (grey) and 630 (orange) nm excitations. Black and red lines indicate the simultaneous target analysis fit. Note that the time axis is linear until 1 ps and logarithmic thereafter. Note also that each panel is scaled to its maximum. Overall rms error of the fit was 87.2 μOD.

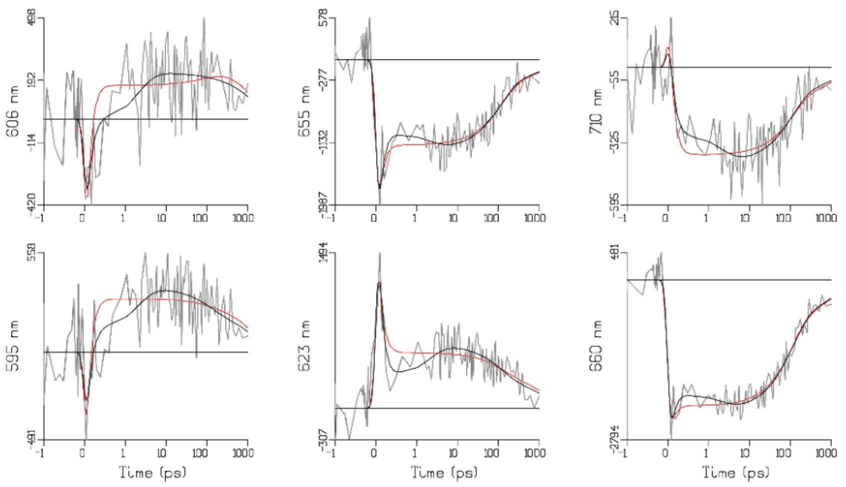

Figure S9. Transient difference absorption of CK-NTD at 6 wavelengths after 694 nm excitation (160 µW). The results of the global analysis of the dataset obtained after excitation at 694 nm, fitting with either 3 (red) and 4 (black) components, on top of raw data (gray lines). A misfit is obtained if the fourth component is not added. The rms error of the fit decreases from 147.4 (with 3 components) to 145.3 (with 4 components). A clear improvement in the fit is visible around 1 ps.

References

(1) Van Stokkum, I. H. M.; Gwizdala, M.; Tian, L.; Snellenburg, J. J.; Van Grondelle, R.; Van Amerongen, H.; Berera, R. A Functional Compartmental Model of the Synechocystis PCC 6803 Phycobilisome. *Photosynth. Res.* **2018**, *135* (1), 87–102.

(2) Tian, L.; Gwizdala, M.; van Stokkum, I. H. M.; Koehorst, R. B. M.; Kirilovsky, D.; van Amerongen, H. Picosecond Kinetics of Light Harvesting and Photoprotective Quenching in Wild-Type and Mutant Phycobilisomes Isolated from the Cyanobacterium Synechocystis PCC 6803. *Biophys. J.* **2012**, *102* (7), 1692–1700.

(3) Tian, L.; Farooq, S.; van Amerongen, H. Probing the Picosecond Kinetics of the Photosystem II Core Complex in Vivo. *Phys. Chem. Chem. Phys.* **2013**, *15* (9), 3146–3154.

(4) Maksimov, E. G.; Schmitt, F.-J.; Shirshin, E. A.; Svirin, M. D.; Elanskaya, I. V; Friedrich, T.; Fadeev, V. V; Paschenko, V. Z.; Rubin, A. B. The Time Course of Non-Photochemical Quenching in Phycobilisomes of Synechocystis Sp. PCC6803 as Revealed by Picosecond Time-Resolved Fluorimetry. *Biochim. Biophys. Acta (BBA)-Bioenergetics* **2014**, *1837* (9), 1540–1547.

(5) Mascoli, V.; Liguori, N.; Xu, P.; Roy, L. M.; van Stokkum, I. H. M. M.; Croce, R. Capturing the Quenching Mechanism of Light-Harvesting Complexes of Plants by Zooming in on the Ensemble. *Chem* **2019**, *5* (11), 2900–2912.

(6) Liguori, N.; Xu, P.; Van Stokkum, I. H. M.; Van Oort, B.; Lu, Y.; Karcher, D.; Bock, R.; Croce, R. Different Carotenoid Conformations Have Distinct Functions in Light-Harvesting Regulation in Plants. *Nat. Commun.* **2017**, *8* (1).

(7) van Stokkum, I. H. M.; Wohlmuth, C.; Würthner, F.; Williams, R. M. Energy Transfer in Supramolecular Calix [4] Arene–Perylene Bisimide Dye Light Harvesting Building Blocks: Resolving Loss Processes with Simultaneous Target Analysis. *J. Photochem. Photobiol.* **2022**, 100154.

(8) Khan, T.; Litvín, R.; Šebelík, V.; Polívka, T.; Polivka, T. Excited State Evolution of Keto-carotenoids after Excess Energy Excitation in the UV Region. *ChemPhysChem* **2021**, *22* (5), 471–480.

(9) Khan, T.; Kuznetsova, V.; Dominguez-Martin, M. A.; Kerfeld, C. A.; Polívka, T. UV Excitation of Carotenoid Binding Proteins OCP and HCP: Excited-State Dynamics and Product Formation. *ChemPhotoChem* **2022**, *6* (1), e202100194.
